# Cognitive and affective control

**DOI:** 10.3389/fpsyg.2012.00477

**Published:** 2012-11-02

**Authors:** Gilles Pourtois, Wim Notebaert, Tom Verguts

**Affiliations:** Department of Psychology, Ghent UniversityGhent, Belgium

Traditionally, cognition and emotion are seen as separate domains that are independent at best and in competition at worst. The French scientist and philosopher Blaise Pascal (1623–1662) famously said “Le coeur a ses raisons que la raison ne connaît point” (The heart has its reasons that reason does not know). Consistent with this quote, many studies in the past have underscored dissociable effects and non-overlapping brain structures of affect and cognition during the control and monitoring of goal-directed behavior (e.g., Bush et al., 2000). Over the last century, however, psychologists and neuroscientists have increasingly appreciated strong reciprocal connections and interactions between cognition and emotion. Initially this was demonstrated in cognitive functions such as perception, attention, learning, memory and decision-making. For instance, an emotional stimulus can alter low-level visual perception (e.g., Bocanegra and Zeelenberg, 2009), and it can capture attention (e.g., Anderson and Phelps, 2001). Likewise, emotional stimuli are better learned and remembered than neutral ones (e.g., McGaugh, 1990) and they can provide strong incentives to bias decision-making (Bechara et al., 1997).

Hence, the independent or competitive view is gradually being replaced by an interactive view. Currently, we focus on interactions of emotion and motivation with cognitive control. Empirical articles and review papers included in this Research Topic timely reveal the extent of overlap and synergistic effects between cognitive control and a wide range of affective processes, both in the normal adult brain, as well as in specific (pathological) conditions, best characterized by either poor or unripe prefrontal-based executive functions as well as impaired affective processes.

Broadly speaking, the original contributions included in this Research Topic tackle one (or more) out of three possible topics. The first and most represented consists of the influence of emotion on cognitive control. Krypotos et al. (2011) focus on the effect of individual differences in emotion regulation, measured by heart rate variability, on response inhibition. van Steenbergen et al. (2011) demonstrate attentional focusing after the presentation of negative pictures. Stürmer et al. (2011) discuss the effect of reward on conflict adaptation. Ridderinkhof et al. (2012) showed that positive affect restored decision learning in patients with Parkinson's disease. Reeck and Egner (2011) demonstrated that irrelevant emotional information distracts more than non-emotional information, supporting affective prioritization in human information processing. Demanet et al. (2011) study the effect of affective stimuli on voluntary task switching. Cavanagh et al. (2011) show that depression is associated with larger error (ERN) signals, suggesting an influence of motivational state on early error processing. Danielmeier and Ullsperger (2011) study the effect of errors (motivationally salient events) on post-error processing. Finally, Chiew and Braver (2011) review the influences of motivational state on early error processing.

In the second category, papers establish a conceptual or anatomical common substrate for cognitive control and emotion. Lowe and Ziemke (2011) endorse a perspective in which emotions are predictions of action tendencies. Aarts et al. (2011) review the literature supporting the hypothesis that (striatal) dopamine regulates the interaction between (appetitive) motivation and cognition. Mushtaq et al. (2011) look at similarities between uncertainty and cognitive control. Mueller (2011) reviews the developmental trajectories of cognitive and emotion control during adolescence. Berggren et al. (2011) emphasize the link between trait-related distractibility in healthy adults and their performance in standard cognitive tasks. Tops and Boksem (2011, 2012) propose that there are two cognitive control systems (one ventral and one dorsal), both of which are partially cognitive and partially affective. Su et al. (2011) propose the glance-look model, specifying how affect and cognitive control interact to produce the attentional blink.

In the third category, a relatively modest number of papers look at the influence of cognitive control on emotion. Krämer et al. (2011) demonstrate a correlation between cognitive control and aggression, suggesting an influence of the former on inhibiting the latter. The paper by Schmidt et al. (2011) reviews the effect of cognitive control on inhibition of thoughts for (being able to) sleep. Paret et al. (2011) demonstrate how cognitive control plays an important role in complex affective processes, such as emotion regulation and the reappraisal of our emotional life. Huizenga et al. (2012) investigate how repeated application of cognitive control influences motivational processing. Finally, the paper by Danielmeier and Ullsperger (2011) investigates the aftereffects of making an error.

In all, the main contribution of this special issue is to highlight similarities and reciprocal influences between cognitive control and emotion. Rather than separate modules, the papers gathered in this special issue concur in suggesting that emotion and cognitive control are two sides of the same coin, as they both contribute, through synergistic effects, to the optimization of behavior. As such, this special issue emphasizes the need to move beyond the classical division or dichotomy between cognitive control and emotion in order to model and account for human goal-directed behavior across various tasks and situations.
